# Supplementary material for: Benchmarking joint multi-omics dimensionality reduction approaches for the study of cancer
Source: Nat Commun. 2021 Jan 5;12:124. doi: 10.1038/s41467-020-20430-7 (PMC7785750; doi:10.1038/s41467-020-20430-7)
Supplement: Supplementary file 1 — Supplementary Information [file 41467_2020_20430_MOESM1_ESM.pdf]

Supp Table 1. Extended list of existing DR multi-omics integrative algorithms. The algorithms are grouped based on their underlying approach. The columns of the table report, the names of the DR method, its underlying approach, the contrained that it assumes on the factors, if it requires to match features and/or samples, the link to the code, the language of the code, if the algorithm has been tested in our benchmark and the link to the paper of the method.

DR approach names	Underlying approach	Constraints on the factors	Dimension matching requirements	Code availability	Language	Tested in Jupyter notebook	Paper citation
**tICA (tensors)**	**Tensorial extension of ICA**	**shared factors**	**matching of both samples and features (tensor)**	**Supplementary Data paper**	**R**	**YES**	**[1]**
tPCA (tensors)	Tensorial extension of PCA	shared factors	matching of both samples and features (tensor)	Supplementary Data paper	R	NO	[1]
PARAFAC (tensors)	Tensorial extension of PCA	shared factors	matching of both samples and features (tensor)	R package multiway	R	NO	[2]
tensor CCA	Tensorial extension of CCA	omics-specific factors	matching of both samples and features (tensor)	https://github.com/rciszek/mdr_tcca	MATLAB	NO	[3]
sCCA	CCA	omics-specific factors	matching of samples	R package PMA	R	NO	[4]
MCCA	CCA	omics-specific factors	matching of samples	NO		NO	[5]
CCA-RLS	CCA	omics-specific factors	matching of samples	NO		NO	[6]
**RGCCA**	**CCA**	**omics-specific factors**	**matching of samples**	**R package RGCCA**	**R**	**YES**	[7]
DIABLO	CCA	omics-specific factors	matching of samples	http://mixomics.org/mixdiablo/	R	NO	[8]
jointNMF	NMF	shared factors	matching of samples	Supplementary Data paper/ MIA on http://page.amss.ac.cn/shihua.zhang/software.html	MATLAB	NO	[10]
MultiNMF	NMF	shared factors	matching of samples	NO		NO	[11]
EquiNMF	NMF	shared factors	matching of samples	NO		NO	[12]
**IntNMF**	**NMF**	**shared factors**	**matching of samples**	**R package intNMF**	**R**	**YES**	**[13]**
iCell	NMF-based matrix tri-factorization	shared factors	matching of samples	http://www0.cs.ucl.ac.uk/staff/natasa/iCell	MATLAB	NO	[14]
**Scikit-fusion**	**Matrix tri-factorization**	**shared factors**	**matching of samples**	**https://github.com/marinkaz/scikit-fusion**	**python**	**YES**	**[15]**
Higher-order GSVD (HO GSVD)	SVD (Matrix tri-factorization)	shared factors	matching of samples	R package hogsvdR	R	NO	[16]
**iCluster**	**Gaussian latent variable model**	**shared factors**	**matching of samples**	**R package iCluster**	**R**	**YES**	[17]
funcSFA	Gaussian latent variable model	shared factors	matching of samples	https://github.com/NKI-CCB/funcsfa	python	NO	[18]
**JIVE**	**Principal Component Analysis (PCA)**	**mixed factors**	**none**	**R package r.jive**	**R**	**YES**	**[19]**
AJIVE	Principal Component Analysis (PCA)	mixed factors	none	https://github.com/MeileiJiang/AJIVE_Project	MATLAB	NO	[20]
**MCIA**	**Co-Inertia Analysis (CIA)**	**omics-specific factors**	**matching of samples**	**R package omicade4**	**R**	**YES**	**[21]**
**MOFA**	**Factor Analysis (FA) (Bayesian)**	**shared factors**	**none**	**https://github.com/bioFAM/MOFA**	**R**	**YES**	**[22]**
Group Factor Analysis (GFA)	Factor Analysis (FA)	shared factors	matching of samples	GFA CRAN package	R	NO	[23]
**MSFA**	**Factor Analysis (FA) (Bayesian)**	**mixed factors**	**matching of samples**	**https://github.com/rdevito/MSFA**	**R**	**YES**	**[24]**
Joint Bayesian factors	Factor Analysis (FA) (Bayesian)	mixed factors	matching of samples	https://sites.google.com/site/jointgenomics/	MATLAB	NO	[25]

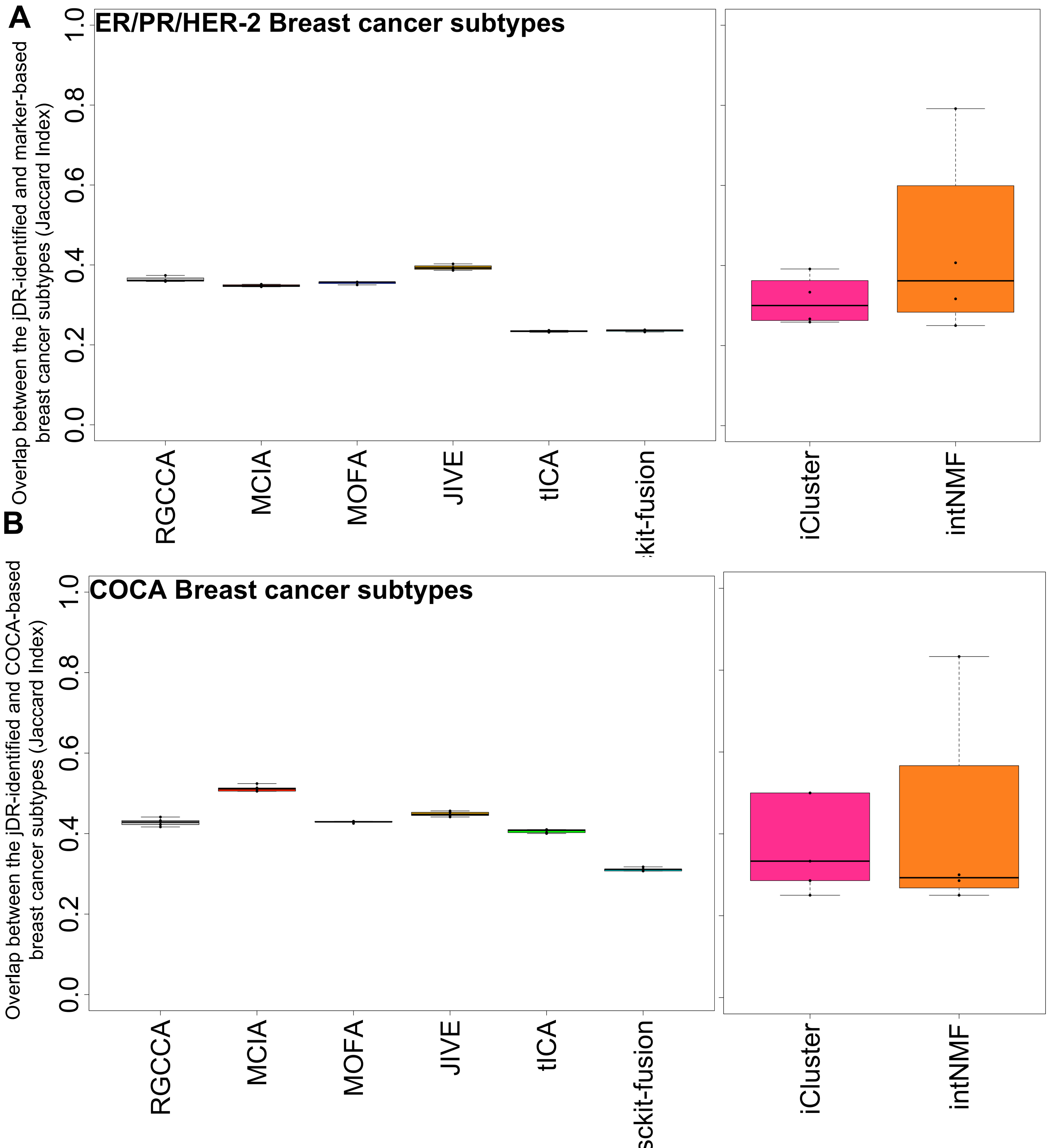

Supp Figure1. Identification of factors associated with breast cancer subtypes by the jDR methods. Boxplots of the Jaccard Index computed between the breast cancer subtypes identified by the different jDR methods and (A) those defined by markers (estrogen receptor, progesterone receptor and HER-2), (B) those defined through COCA clustering by the TCGA Consortium. The number of samples here considered is 621 and the results are obtained over 1000 independent runs of k-means clustering. Data are presented as mean values +/- sd.

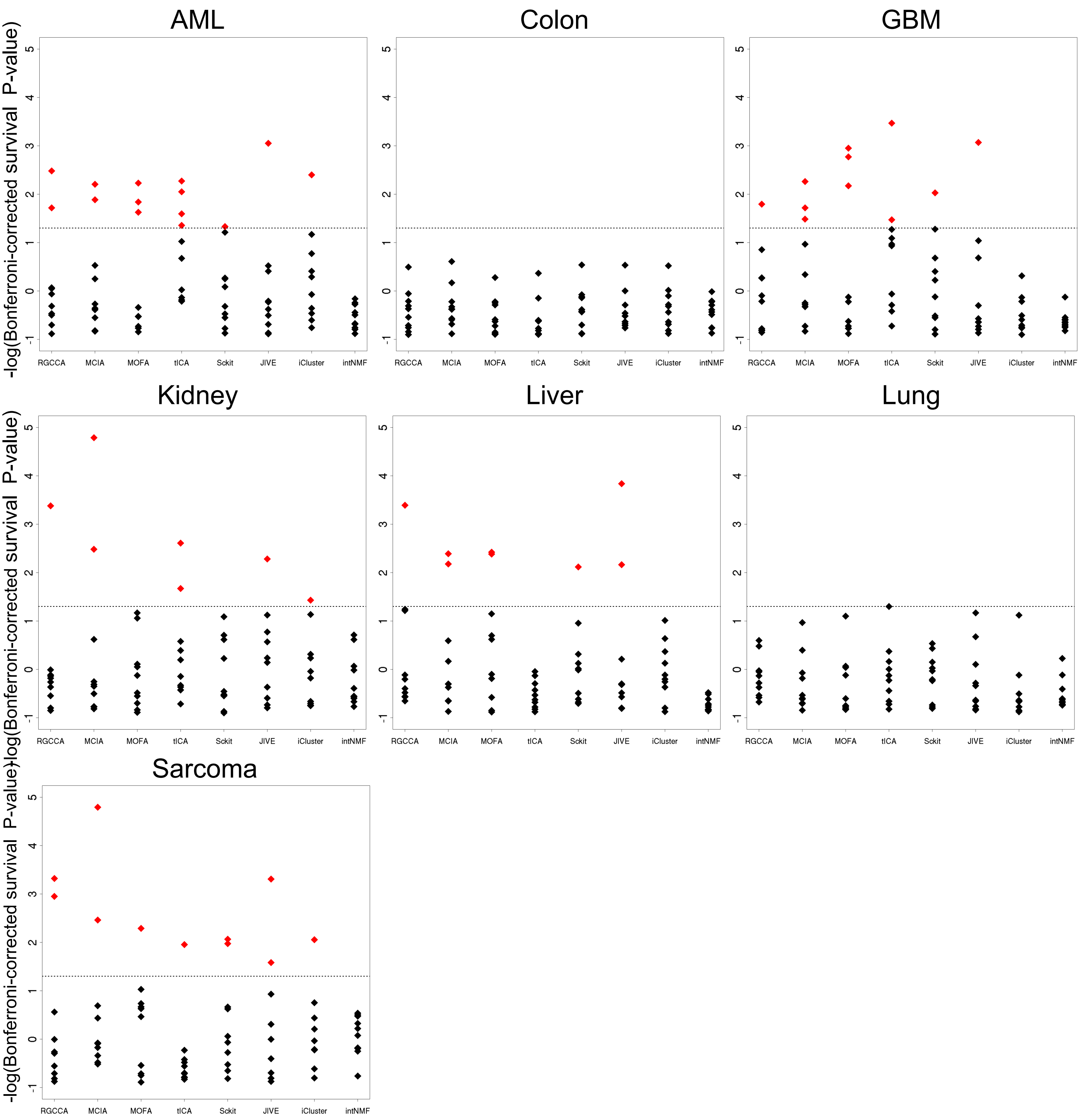

Supp Figure2. Identification of factors predictive of survival in cancer by the jDR methods. For each method the Bonferroni-corrected p-values associating each of the 10 factors to survival (Cox regression-based survival analysis) are reported. The dot lines correspond to a corrected p-value threshold of 0.05.

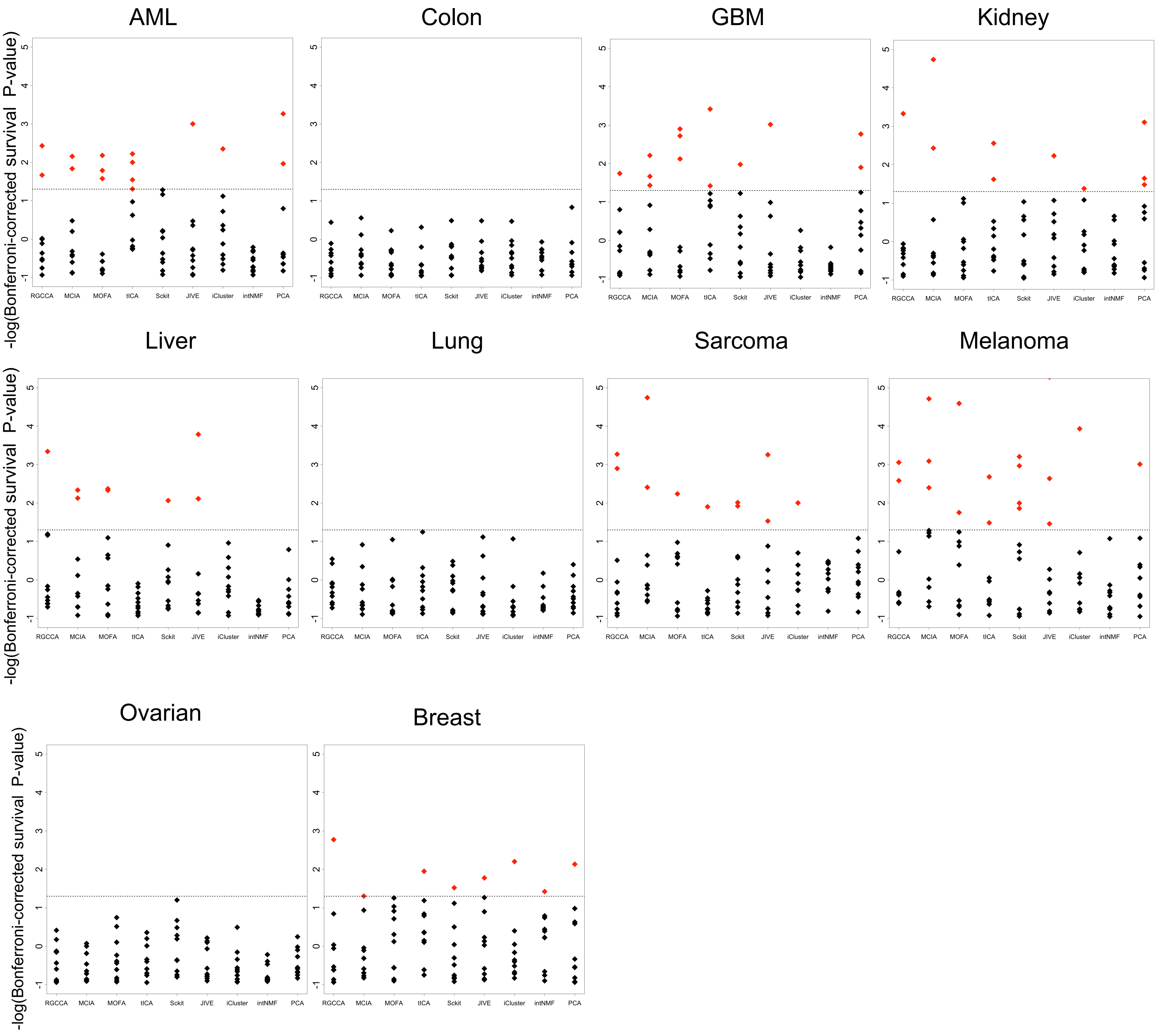

Supp Figure3. Identification of factors predictive of survival in cancer by the jDR methods vs. PCA on transcriptome alone. For each method the Bonferroni-corrected p-values associating each of the 10 factors to survival (Cox regression-based survival analysis) are reported. The dot lines correspond to a corrected p-value threshold of 0.05.

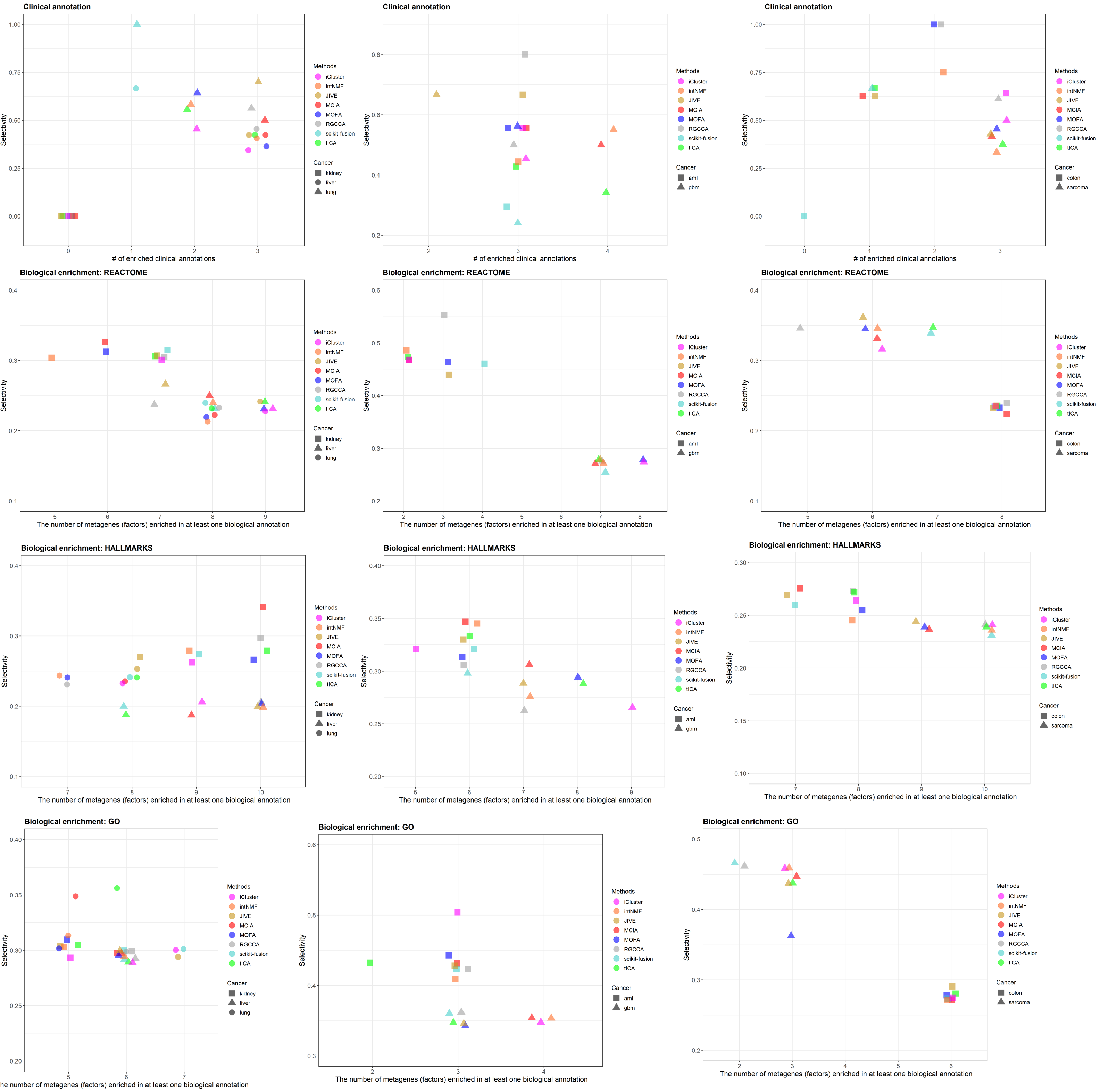

Supp Figure 4. Identification of factors associated with clinical annotations, and metagenes associated with biological annotations in cancer samples, by the jDR methods. For clinical annotations, the plot represents, for each method, the number of clinical annotations enriched in at least one factor together with the selectivity of the associations between the factors and the clinical annotations (Method). For the three annotation sources (MsigDB Hallmarks, REACTOME and Gene Ontology), the number of metagenes identified by the different jDR methods enriched in at least a biological annotation are plotted against the selectivity of the associations between the metagene and the annotation.

References

1. Teschendorff, Andrew E., et al. Genome biology 19.1 (2018): 76.
2. Harshman, Richard A.,et al. Computational Statistics & Data Analysis 18.1 (1994): 39-72.
3. Luo, Yong, et al. IEEE transactions on Knowledge and Data Engineering 27.11 (2015): 3111-3124.
4. Witten, Daniela M., et al. Biostatistics 10.3 (2009): 515-534.
5. Witten, Daniela M., et al. Statistical applications in genetics and molecular biology 8.1 (2009): 1-27.
6. Vía, Javier, et al. Neural Networks 20.1 (2007): 139-152.
7. Tenenhaus, Arthur, et al. Biostatistics 15.3 (2014): 569-583.
8. Singh, Amrit, et al. Bioinformatics (2019).
9. Zhang, Shihua, et al. Nucleic acids research 40.19 (2012): 9379-9391.
10. Liu, Jialu, et al. Proceedings of the 2013 SIAM International Conference on Data Mining.
11. Hidru, Daniel, and Anna Goldenberg.arXiv preprint arXiv:1409.4018 (2014).
12. Chalise P and Fridley B (2017). PLOS ONE, 12(5), e0176278.
13. Malod-Dognin, Noël, et al. Nat comm 10.1 (2019): 805.
14. Žitnik, Marinka, and Blaž Zupan. "Data fusion by matrix factorization." IEEE transactions on pattern analysis and machine intelligence 37.1 (2015): 41-53.
15. Sankaranarayanan, Preethi, et al. PloS one 6.12 (2011): e28072.
16. Shen, Ronglai, et al. PloS one 7.4 (2012): e35236.
17. Bismeijer, Tycho et al. PLoS computational biology 14.10 (2018): e1006520.
18. Lock, Eric F., et al. The annals of applied statistics 7.1 (2013): 523.
19. Feng, Qing, et al. Journal of Multivariate Analysis 166 (2018): 241-265
20. Meng, Chen, et al. BMC bioinformatics 15.1 (2014): 162.
21. Argelaguet, Ricard, et al. Molecular systems biology 14.6 (2018): e8124.
22. Leppäaho, E. et al. The Journal of Machine Learning Research 18.1 (2017): 1294-1298.
23. De Vito, Roberta, et al. arXiv preprint arXiv:1611.06350 (2016).
24. Ray, Priyadip, et al. "Bayesian joint analysis of heterogeneous genomics data." Bioinformatics 30.10 (2014): 1370-1376.
