# Supplementary material for: Benchmarking joint multi-omics dimensionality reduction approaches for the study of cancer
Source: Nat Commun. 2021 Jan 5;12:124. doi: 10.1038/s41467-020-20430-7 (PMC7785750; doi:10.1038/s41467-020-20430-7)
Supplement: Supplementary file 3 — Reporting Summary [file 41467_2020_20430_MOESM3_ESM.pdf]

nature research

Corresponding author(s): Laura Cantini and Anais Baudot

Last updated by author(s): Nov 6, 2020

Reporting Summary

Nature Research wishes to improve the reproducibility of the work that we publish. This form provides structure for consistency and transparency in reporting. For further information on Nature Research policies, see our Editorial Policies and the Editorial Policy Checklist.

Statistics

For all statistical analyses, confirm that the following items are present in the figure legend, table legend, main text, or Methods section.

n/a	Confirmed	
☐	☒	The exact sample size (*n*) for each experimental group/condition, given as a discrete number and unit of measurement
☐	☒	A statement on whether measurements were taken from distinct samples or whether the same sample was measured repeatedly
☐	☒	The statistical test(s) used AND whether they are one- or two-sided<br>*Only common tests should be described solely by name; describe more complex techniques in the Methods section.*
☒	☐	A description of all covariates tested
☐	☒	A description of any assumptions or corrections, such as tests of normality and adjustment for multiple comparisons
☒	☐	A full description of the statistical parameters including central tendency (e.g. means) or other basic estimates (e.g. regression coefficient) AND variation (e.g. standard deviation) or associated estimates of uncertainty (e.g. confidence intervals)
☐	☒	For null hypothesis testing, the test statistic (e.g. *F*, *t*, *r*) with confidence intervals, effect sizes, degrees of freedom and *P* value noted<br>*Give P values as exact values whenever suitable.*
☒	☐	For Bayesian analysis, information on the choice of priors and Markov chain Monte Carlo settings
☒	☐	For hierarchical and complex designs, identification of the appropriate level for tests and full reporting of outcomes
☒	☐	Estimates of effect sizes (e.g. Cohen's *d*, Pearson's *r*), indicating how they were calculated

Our web collection on statistics for biologists contains articles on many of the points above.

Software and code

Policy information about availability of computer code

Data collection	All input data used in the paper are public. The simulated data are produced using the R package InterSIM V2.2. The cancer TCGA data were dowloaded from http://acgt.cs.tau.ac.il/multi_omic_benchmark/download.html. The single-cell data are available in the data/ folder of our github repository (https://github.com/ComputationalSystemsBiology/momix-notebook). Annotations (i.e., Gene Ontology, Reactome and MSigDB Hallmarks) were fetched from fgsea V1.16.
Data analysis	All the analyses can be reproduced with the momix Jupyter notebook available at https://github.com/ComputationalSystemsBiology/momix-notebook. The same github link also contains the instructions to install the momix conda environment available on Anaconda cloud (https://anaconda.org/lcantini/momix). With this conda environment, the user will get all the required R and Python packages automatically installed, in the same version that we used for this benchmark. The packages used are: R packages (iCluster V2.1, intNMF V1.2, r.jive V2.1, omicade4 V1.3, MOFA V1, MSFA V1, RGCCA2.1.2, InterSIM V2.2, fgsea V1.16). Python packages (scikit-fusion V1).

For manuscripts utilizing custom algorithms or software that are central to the research but not yet described in published literature, software must be made available to editors and reviewers. We strongly encourage code deposition in a community repository (e.g. GitHub). See the Nature Research guidelines for submitting code & software for further information.

Data

Policy information about availability of data

All manuscripts must include a data availability statement. This statement should provide the following information, where applicable:

- Accession codes, unique identifiers, or web links for publicly available datasets
- A list of figures that have associated raw data
- A description of any restrictions on data availability

The simulated data are produced using the R package InterSIM V2.2. The cancer TCGA data were dowloaded from http://acgt.cs.tau.ac.il/multi_omic_benchmark/

download.html. The single-cell data are available in the data/ folder of our github repository (https://github.com/ComputationalSystemsBiology/momix-notebook). Annotations (i.e., Gene Ontology, Reactome and MSigDB Hallmarks) were fetched from fgsea V1.16.

Field-specific reporting

Please select the one below that is the best fit for your research. If you are not sure, read the appropriate sections before making your selection.

- [x] Life sciences
- [ ] Behavioural & social sciences
- [ ] Ecological, evolutionary & environmental sciences

For a reference copy of the document with all sections, see nature.com/documents/nr-reporting-summary-flat.pdf

Life sciences study design

All studies must disclose on these points even when the disclosure is negative.

Sample size	No dataset has been produced in this study. For simulated data, we simulated one dataset per condition, and focused on the effect of different cluster numbers and sizes. The number of samples is 100 samples per dataset. For cancer data, we used TCGA datasets from 10 cancer types. Each cancer dataset contains 100-1000 samples. Finally, for single-cell analyses, we used the only dataset containing cancer cells jointly profiled for scATAC-seq and scRNA-seq (206 cells).
Data exclusions	Public available data used. No sample and no data have been excluded from the analysis.
Replication	All results can be replicated through the Jupyter Notebook https://github.com/ComputationalSystemsBiology/momix-notebook and its associated momix conda environment available on Anaconda cloud.
Randomization	The study does not involve data collection
Blinding	The study does not involve data collection

Reporting for specific materials, systems and methods

We require information from authors about some types of materials, experimental systems and methods used in many studies. Here, indicate whether each material, system or method listed is relevant to your study. If you are not sure if a list item applies to your research, read the appropriate section before selecting a response.

Materials & experimental systems

n/a	Involved in the study
[x]	[ ] Antibodies
[x]	[ ] Eukaryotic cell lines
[x]	[ ] Palaeontology and archaeology
[x]	[ ] Animals and other organisms
[x]	[ ] Human research participants
[x]	[ ] Clinical data
[x]	[ ] Dual use research of concern

Methods

n/a	Involved in the study
[x]	[ ] ChIP-seq
[x]	[ ] Flow cytometry
[x]	[ ] MRI-based neuroimaging
